# Relationship between Enterprise Financing Structure and Business Performance Assisted by Blockchain for Internet of Things Financing Mode

**DOI:** 10.1155/2022/2076830

**Published:** 2022-05-31

**Authors:** Jing Li, Hao Feng, Mao Li, ManJie Li, Yuyuan Chen

**Affiliations:** Information Engineering Institute, Wuhan College, Wuhan 430212, Hubei, China

## Abstract

Financing structure is an important and very complex issue in the financial theory and the rights and obligations of relevant stakeholders of enterprises are also concentrated in the financing structure. Therefore, the financing structure has a significant impact on the value of enterprises. A reasonable financing structure is conducive to standardizing the behavior of enterprises and improving the value of enterprises. The change of corporate financing structure is often used as a signal to external investors about the company's future income expectations, especially because the financing structure has a certain impact on the company's performance, which makes the problem of financing structure more valued by the theoretical and financial circles. For the empirical information about company financing, this paper explores the influencing elements of the company's running overall performance assisted with the aid of the blockchain, and the net of matters provides a chain model and constructs the operating performance indicators according to the comprehensive score. We select the commercial credit financing rate, short-term loan financing rate, long-term loan financing rate, debt financing rate, equity financing rate, and endogenous financing rate. The control variables are total capital, ownership concentration, and average age of the company. The conclusion is drawn by regression analysis. Commercial savings financing rate, fairness financing fee, and endogenous financing fee are positively correlated with the working performance; short-term loans and average age of the company are negatively correlated with the operating performance; and long-term loan financing rate, bond financing rate, and equity concentration are not significantly correlated with the operating performance.

## 1. Introduction

The problem of difficult and expensive financing of enterprises has always been the concern of the industry. Research and practice show whether it is caused by the enterprise's own genes, such as small scale, insufficient collateral, high business risk, short life cycle, and weak ability to resist risk or because there are still some deficiencies in the financial services provided by the current financial system, and financial institutions are unwilling to bear risk losses and lend funds to enterprises with small returns, which leads to the difficulty of enterprise financing, which are all practical problems existing in the process of enterprise financing [1]. The solution mode usually adopts supply chain financing, financial institution financing, intellectual property pledge financing, equity crowdfunding financing, venture capital, and so on. Scholars have analyzed these models and obtained the limitations of different models from different aspects [2]. For example, intellectual property pledge financing can effectively deal with enterprise financing, but there must be a smooth operation mechanism. The projects initiated by enterprises and the growth of projects have an important impact on equity crowdfunding financing, which can be used for reference to help enterprises obtain financing [3]. However, there are problems such as long review time before loan and asymmetric information of project implementation after loan. Venture capital can attract a large amount of private capital to the Chinese enterprises, but it is faced with the lack of docking platform, poor management, and low exit efficiency.

The development of blockchain and IOT technology has attracted great attention from all walks of life. Blockchain is a new technology that has been rising in recent years. It has the characteristics of decentralization, traceability, programmability, high security, and high credibility [4]. It is expected to reveal the real information of enterprises; improve the trust of both investors and financiers; solve the problems of difficult, slow, and expensive financing; and improve the efficiency of financing [5]. The Internet of things technology is the third information wave of the development of computer and Internet. It is the product of the fourth industrial revolution and represents the development trend of the next generation of information technology. The most prominent feature of the Internet of things is intelligence; it uses the advantages of the Internet of things to connect things, to uniquely identify and manage the goods to be pledged by enterprises, and realize the intelligent management of enterprises, so as to improve the efficiency of enterprises and liberate labor [6]. It promotes the development of enterprises by giving full play to the unique communication principle and by combining with the traditional service industry [7]. The Internet of things technology is the expansion and extension of the Internet. With the help of the Internet of things technology, new changes can be made to the financing mode and financing environment of enterprises, so that enterprises can “supply what they need” in reality [8]. Therefore, the financing problem of enterprises can be solved with the help of blockchain and IoT finance. However, these studies did not make an in-depth and systematic analysis on how blockchain and IoT solve the core problem of enterprise financing.

Based on the analysis of the operation mechanism of blockchain and IoT financing, this paper intends to study the enterprise's blockchain and IoT supply chain financing mode from the perspective of the comparison of blockchain and IoT systems, trying to provide solutions to the enterprise's financing problems. The chapters of this paper are arranged as follows: Section 1 is the introduction. This paper expounds the research background, purpose, and significance of this paper. Related work is discussed in Section 2.  Section 3 expounds the relevant knowledge of the supply chain financing mode of blockchain and IoT.  Section 4 analyzes the current situation of the financing structure and business performance of enterprises assisted by the blockchain and IoT supply chain financing mode.  Section 5 makes an empirical analysis on the relationship between enterprise financing structure and business performance assisted by blockchain and IoT supply chain financing mode.  Section 6 summarizes the full text and further finds out the shortcomings and limitations of this paper in the empirical research.

## 2. Related Work

The relationship between financing structure and business performance has been widely discussed and analyzed. Foreign scholars believe that studying the rationality of the financing structure is of great significance to enterprise management. At present, scholars have not reached a conclusion on the way to reasonably adjust the financing structure, but through continuous assumptions and verification, a reasonable financing structure will be helpful to improve the business performance of enterprises. The proportion of the financing structure to the operating performance of an enterprise refers to the proportion of its liabilities to its operating performance. Taking rail transit enterprises as the starting point, this paper analyzes the relationship between their financing structure and business performance. There are many theories about financing structure at home and abroad, which provides a reference for relevant theoretical research and practical treatment. The above literature can be summarized as follows:

Most scholars at home and abroad pay attention to the degree of ownership concentration and on the basis of it, China has added the nature of ownership to analyze the impact of ownership and business performance [9]. In short, the purpose of academic research is to reasonably optimize the equity financing structure of enterprises, significantly promote the improvement of business performance, and ensure the sustainable, orderly, and healthy development of enterprises [10]. The depth and breadth of foreign theoretical research on the financing structure far exceeds that of domestic scholars, and there is still a great lack of domestic research: first, domestic scholars are limited to discussing the impact of financing structure or corporate performance, they do not further explore the internal causes of empirical analysis results, and stop at the universal theoretical reasons behind the research problems [11]. If we only study the impact of financing structure on corporate performance from one aspect, the conclusion will be insufficient [12]. Second, domestic scholars did not conduct in-depth research on the optimization of the financing structure of an industry and put forward effective suggestions. They only analyzed whether there was a positive and negative relationship between the financing structure and business performance, and the significance of the analysis results to stakeholders is limited [13].

The early research of domestic and foreign scholars mainly focused on the relationship between financing structure and business performance, and the conclusions are not unified [14]. When enterprises make financing decisions, most shareholders with low shareholding ratio only pay attention to whether the company operates steadily and meets the expected profits, but pay little attention to the development of enterprises and do not give full play to the role of decision makers [15]. However, when corporations finance externally, it will make businesses make prudent selections on capital use, enterprise improvement, and income administration mode, which has a nice effect on commercial enterprise's overall performance [16]. According to the different ways of enterprise capital integration, a stable capital system will drive the optimization of the company's capital value and help to achieve the business objectives of maximizing the enterprise value and profit [17]. Other enterprises can also learn from this and promote their own development through debt financing.

## 3. Relevant Knowledge of IoT Supply Chain Financing Mode

Using blockchain and IoT technology, on the one hand, can record the data flow between various types of institutions in the financing process, effectively improve the transparency of financial information and the traceability of data, and improve enterprise credit, so as to solve the problems such as the difficulty of solving the financing trust crisis in the financing process [18]. On the other hand, the Internet of things technology realizes the information connection between people and things, so as to realize the real-time connection of goods information in the real world, which can greatly alleviate the problem of information asymmetry. Meanwhile, using RFID can uniquely identify and manage the pledged goods of enterprises and supervise the goods.

### 3.1. Internet of Things Supply Chain Financing Model

**Figure 1:**
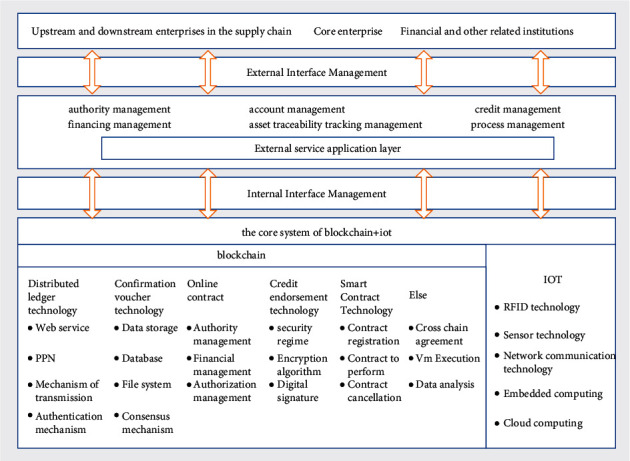
Basic framework of blockchain for the Internet of things financing model.

Limited by the factors of small scale and low credit rating, enterprises cannot guarantee the safety and income of funds, so most financial institutions are reluctant to lend funds to them, so that they cannot get normal supply when enterprises urgently need funds. The Internet of things forms a database for the information transmission and processing of various goods of enterprises, and finally forms an Internet of things financing platform [16]. Through the interface of the Internet of things financing platform, enterprises, financial institutions, and investors can clearly understand the current temporal and spatial state of goods and alleviate the transmission asymmetry of information of all parties [17]. Financing under the Internet of things, when enterprises borrow from borrowers, the information sources that borrowers can collect are no longer a single connection, but resource allocation and information sharing in all aspects from raw material production to products and goods in transit [18]. For example, with the help of the Internet of things platform, there is more intersection of information links between enterprises, borrowers, and sellers. Supply chain financing is no longer a single simple financing line cantered on core enterprises but extends to the interactive links between multiple core enterprises, upstream and downstream enterprises, and multiple supply chains. The basic framework of blockchain and IoT supply chain financing model is shown in Figure 1.

As shown in Figure 1, upstream and downstream enterprises like suppliers and distributors, core enterprises, finance, and other relevant institutions manage authority, account, credit, financing, asset traceability, and process through the blockchain and IoT system; leave traces of the data generated by various enterprises and business links in the blockchain network; and uniquely identify and manage the pledged goods.

**Figure 2:**
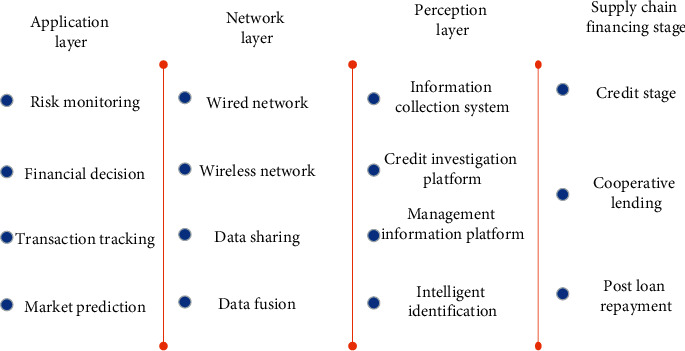
Basic framework of the IoT supply chain financing model.

The Internet of things is composed of four layers: application layer, data processing layer, network transmission layer, and perception layer [19]. Among them, the perception layer is the intelligent perception and recognition of the electronic tag of the cargo information, scanning the information, and uploading it. We use the local network transmission layer to process and analyze the collected data by means of wireless or wired and then collect and transmit the corresponding data. Finally, it can realize a variety of intelligent applications of the Internet of things system [20]. Internet of things mode financing is to upload the goods' information to the data supervision platform through the perception layer, so as to realize the intelligent positioning, tracking, monitoring, and intelligent management of a variety of “in transit, in warehouse, and in processing” real goods; form an Internet of things database; form a financing platform through the centralized processing of goods, enterprise information, risk, and other data; and connect several system interfaces to realize overall management. Through the interface of this platform, enterprises, investors, supervisors, and other participants can accurately locate and supervise the information and location of goods from the perspective of time and space and fully perceive the changes of their transaction information and other data. Its operation framework is shown in Figure 2.

### 3.2. Current Situation of Supply Chain Financing Mode of Blockchain for Internet of Things

**Figure 3:**
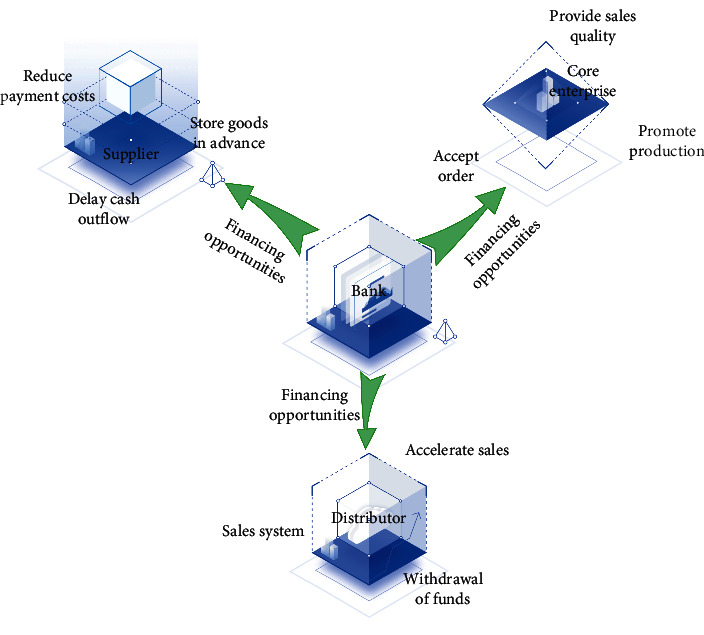
Relationship between the elements of blockchain for the Internet of things supply chain financing model.

Internet of things supply chain financing mode is a new financing mode in which borrowing institutions take core enterprises as the center and provide financing to the upstream, middle, and downstream enterprises. It not only solves the dilemma of the shortage of traditional enterprise financing channels but also makes banks and enterprises more closely linked. The bank judges whether to provide loans to its upstream, middle, and downstream enterprises according to the credit capacity of the core enterprises in the chain. Its development is the outsourcing of production and operation business, and the production of products is generated from the supply chain inside the enterprise to outside the enterprise, which promotes the birth of IoT supply chain financing to reduce capital flow for the production of products [21]. The application of IoT supply chain financing is different from bank credit. It mainly focuses on the high-quality projects of enterprises, takes the core enterprises as the center, and extends the credit system to the upstream and downstream enterprises associated with it. The relationship between the elements of the Internet of things supply chain financing model is shown in Figure 3.

### 3.3. Enterprise Application of Blockchain for Internet of Things Financing

The use of blockchain for the Internet of things technology enables complete business information recording and backtracking, enterprises applying for loans, the intelligent positioning of goods, and many data such as transaction information, enterprise operation status, and capital flow can be uploaded to blockchain for the Internet of things financing platform in real time. Through blockchain for the Internet of things financing platform, enterprises release financing needs on the platform, while investors master the verification level, disclosure information, risk prediction, and other information of enterprises through the platform, so as to realize the connection between demand and supply and avoid the risk of staying and misappropriating funds of previous Internet financing platforms. We can know the state of the pledge all the time, avoid the disadvantages of pooling of the pledge and the untimely transmission of the information of the pledge, and provide a convenient financing mode for the development of enterprises.

## 4. Analysis on the Current Situation of Enterprise Financing Structure and Performance

According to the data, first, it arranges and analyzes the current situation of business performance and financing structure, in order to avoid unnecessary errors as much as possible and affect the authenticity of the results.

### 4.1. Analysis on the Current Situation of Financing Structure

The analysis of financing structure on business performance has always been favored by researchers. Internal financing, equity financing, and debt financing constitute the financing structure. In order to understand the financing structure more finely, we need to dissect the financing structure carefully.

**Figure 4:**
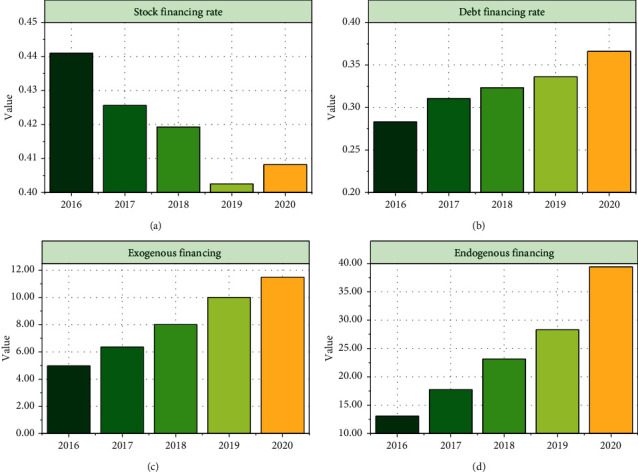
Analysis on the current situation of the financing structure.

As can be seen from Figure 4(a), enterprises assisted by blockchain for Internet of things supply chain financing mode still prefer external financing. The amount of endogenous financing increased from an average of 497.86 million yuan in 2016 to an average of 1150.6 million yuan in 2020. The amount is increasing, but the proportion is shrinking. This shows that the share of endogenous financing in the financing structure is decreasing, and more people begin to choose external financing. External financing increased from an average of 1.30663 billion yuan in 2016 to an average of 3.94321 billion yuan in 2020, with rapid variable growth. As can be seen from Figure 4(b), the average value of undistributed profits within five years is 0.0798, 0.0802, 0.0812, 0.0840, and 0.0885, respectively. The average value of undistributed profits of enterprises assisted by blockchain for Internet of things supply chain financing mode is relatively stable. However, the variance change is larger than that of accumulated depreciation and surplus reserve. The role of variance and standard deviation depends on the dispersion of data. It can be seen from the table that surplus reserve is the most stable component in the endogenous financing structure. The second is accumulated depreciation, and the last is undistributed profit. As can be seen from Figure 4(c), the average equity proportion of enterprises assisted by blockchain for Internet of things supply chain financing mode in recent five years is 0.0828, 0.0924, 0.0896, 0.0931, and 0.0953, respectively. It can be seen that the proportion of equity is steadily increasing. The proportion of capital reserve has been relatively stable in the recent five years, but it can be seen from the variance of the two in recent five years that the dispersion of the proportion of capital reserve is greater than that of the variance. It can be seen from Figure 4(d) that from 2016 to 2020, short-term loan financing has become the most important component, but according to the gap between the maximum and minimum in recent five years, the maximum is 2018, the maximum is 0.5467, and the minimum is 0. The variance and standard deviation of the average value are also the largest in the composition of the debt financing structure. The average value of variance is 0.0087 and the average value of standard deviation is 0.0933. Compared with other debt financing structures, the average value of standard deviation is the largest. According to the significance of variance and standard deviation, the difference and change of short-term loan financing methods in enterprises are the largest.

### 4.2. Analysis on the Current Situation of Business Performance

We select the financial indicators of profitability, operation ability, solvency, and development ability to analyze the current situation of enterprise performance.

#### 4.2.1. Analysis of the Enterprise Profitability

**Figure 5:**
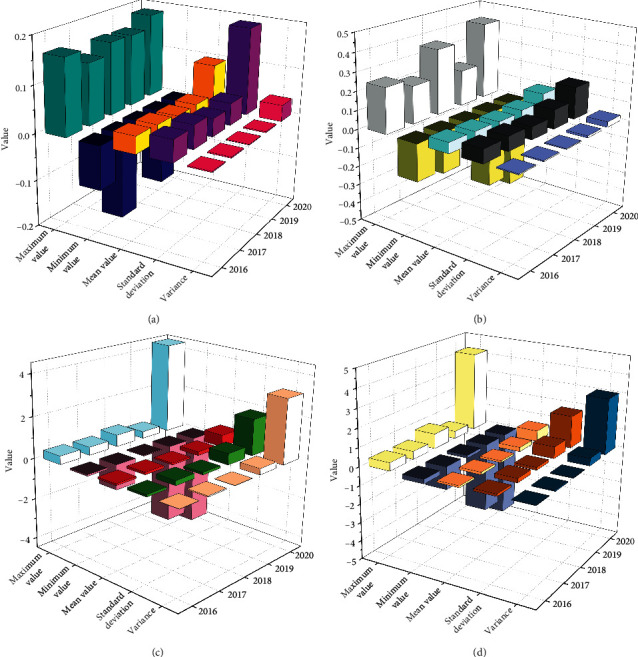
Profitability and earnings quality data of enterprises. (a) Total property return rate. (b) Return on net assets. (c) Retained profits rate. (d) Operating margin.

From the perspective of its actual profitability and income quality indicators, the profitability and income quality data of enterprises assisted by blockchain for Internet of things supply chain financing model are shown in Figure 5.

As can be seen from Figure 4, the rate of return on total assets of the enterprise decreases first and then increases, indicating that the level of input and output of the enterprise increases again. However, the fly in the ointment is that according to the variance of the rate of return on total assets, it can be concluded that the dispersion degree of the enterprise is increasing, that is, there are more and more two-level differentiation in the profitability of the enterprise. According to the profit margin of net assets, it can be analyzed that the after-tax profitability of enterprises assisted by the supply chain financing mode of blockchain for Internet of things will increase steadily from 2016 to 2020, especially in the second two years, but at the same time, the differentiation of enterprises is similar to the return on total assets. Through costs, the net interest rate is a measure of the cost paid by enterprises to obtain benefits, and the two are inversely proportional. The number of enterprises assisted by blockchain for Internet of things supply chain financing mode decreased first and then increased and the proportion increased rapidly. It is possible that the enterprise's ability to control costs will weaken again.

#### 4.2.2. Analysis on the Solvency of Enterprises

**Figure 6:**
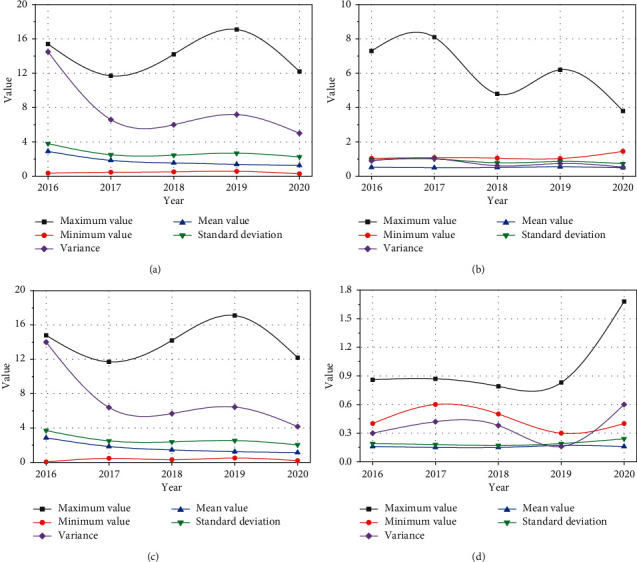
Analysis on solvency index of enterprises. (a) Current ratio. (b) Equity multiplier. (c) Quick ratio. (d) Asset liability ratio.

In order to study the solvency of enterprises assisted by blockchain for Internet of things supply chain financing mode, four important indicators are selected. The data are shown in Figure 6.

As can be seen from Figure 5, the broken line chart of enterprise solvency assisted by blockchain for Internet of things supply chain financing mode shows that the asset liability ratio of enterprises assisted by blockchain for Internet of things supply chain financing mode is almost stable at about 16%. It shows that the debt and loan of listed companies in this industry only accounts for about 16% of the total assets. From the variance, we can see that it is almost stable. Except for a little increase in 2019, it remains at about 3.5% every year. It shows that the whole industry is still relatively stable. Current ratio and quick ratio are selected for short-term borrowings. The ideal value of the current ratio is 2, and the lower limit of the current ratio is 1. If the current ratio is too low, it means that the enterprise is difficult to repay on schedule. If it is too high, it means that the occupation of current assets is too high, which will affect the use of funds and profitability of the enterprise.

#### 4.2.3. Analysis of the Enterprise Operation Capacity

**Figure 7:**
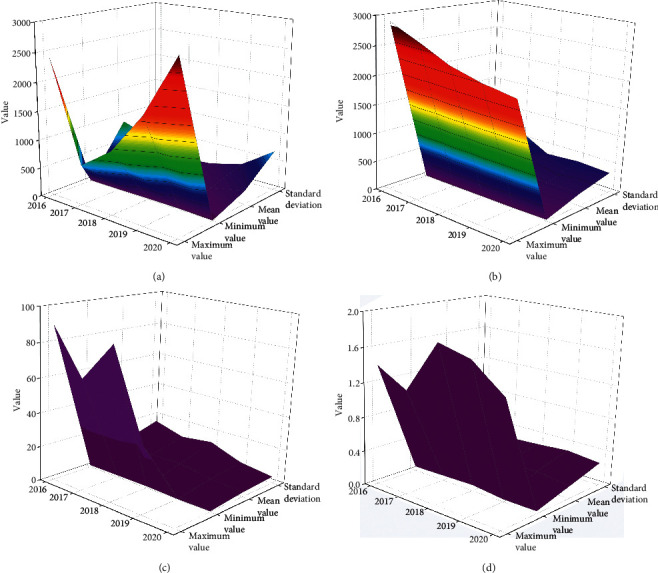
Analysis of enterprise operation capacity. (a) Inventory turnover. (b) Business cycle. (c) Cycle rate of accounts receivable. (d) Total asset turnover.

The operating capacity of enterprises assisted by blockchain for Internet of things supply chain financing mode is shown in Figure 7.

If the inventory turnover rate is too low, it will lead to the interruption of reproduction or tight sales, but it is also easy to form product backlog if there is too much storage. The normal production and operation can only be ensured if the inventory turnover rate is in a reasonable range. Through the annual data feedback from 2016 to 2020, it can be concluded that the enterprise is moving from a too high inventory turnover rate to a reasonable range, indicating that the enterprise's sales capacity, all aspects of operation capacity, and inventory management level continue to improve. Business cycle is an important factor to measure the current assets of enterprises, the shorter the business cycle, the faster the capital turnover. The data have been stable and small, the operation ability of the enterprise has been improving continuously, and the overall management has been effective. The turnover rate of accounts receivable is an index to measure the collection speed of an enterprise. The higher the turnover rate of accounts receivable, the shorter the accounting period of the enterprise and the faster the return of funds.

#### 4.2.4. Analysis on the Growth Ability of Enterprises

**Figure 8:**
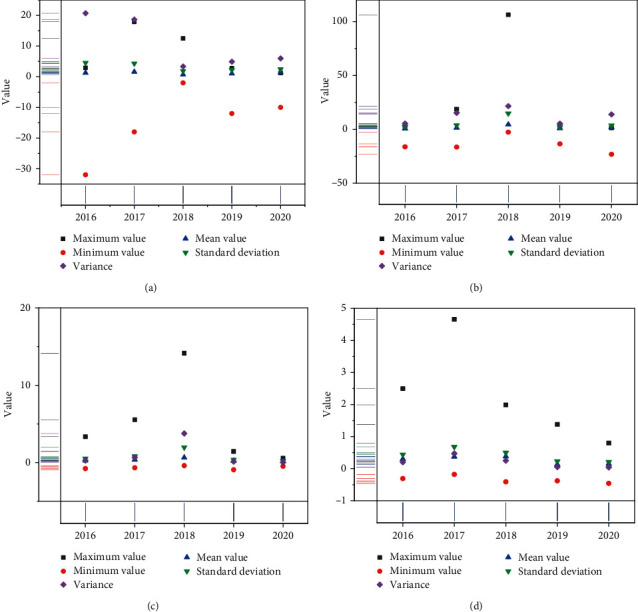
Analysis on growth capability index of enterprises. (a) Net profit growth rate. (b) Total profit increased year-on-year. (c) Total operating revenue increased year-on-year. (d) Growth rate of total asset.

The analysis of the enterprise growth ability is the prediction of the future development trend and development speed of the enterprise and the analysis of the expansion of the business ability of the enterprise. The growth capability indicators of enterprises assisted by blockchain for Internet of things supply chain financing mode are shown in Figure 8.

Overall, except for the highest year-on-year growth of total profit in 2017, it has slowed down in other years. However, from the perspective of net profit, the profit is not very high, which is directly proportional to it. It shows that the company continues to expand. On the whole, the profit growth of enterprises is not very stable. It can be seen from the picture that the total assets of the enterprise continue to expand.

## 5. Empirical Analysis

In this paper, the multiple linear regression model is based on multiple financial indicators, so the degree of multicollinearity of these variables is statistically tested before analysis using the multicollinearity test function of SPSS software, the tolerance, and variance expansion factor (VIF) are calculated. If the VIF value of each variable is less than 10 (or the tolerance is greater than 0.1), it is considered that there is no multicollinearity between variables, which is suitable for the next statistical analysis.

### 5.1. Relationship between the Overall Financing Structure and Enterprise Performance

**Figure 9:**
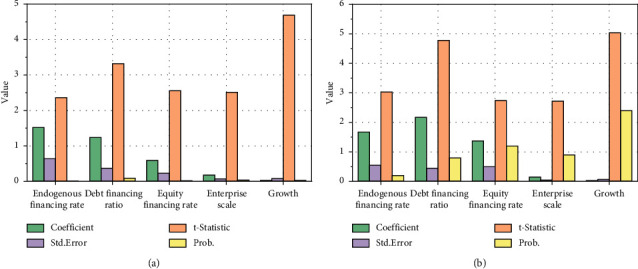
Multiple regression results of financing structure on enterprise performance. (a) Individual fixed effect model. (b) Random effect model.

We establish the individual fixed effect model of the overall financing structure on enterprise performance. The specific multiple regression results are shown in Figure 9.

As can be seen from Figure 8, the regression coefficient of endogenous financing rate is 1.673396, and the test value with significant coefficient difference is 0.0026. There is a significant positive correlation between endogenous financing level and enterprise performance at the level of 1%. This shows that endogenous financing has a positive impact on the improvement of enterprise performance of China's petroleum and petrochemical enterprises, which is consistent with the priority financing theory. When enterprises need to raise funds, they will give priority to financing through endogenous financing. The regression coefficient of debt financing ratio is −2.179861, and the test value with significant coefficient difference is 0. The scale of debt financing is significantly negatively correlated with enterprise performance at the level of 1%. The debt financing scale of petroleum and petrochemical enterprises is relatively large, and the average debt financing rate is 52.08%. The debt level of enterprises is relatively high, and the financial risk of enterprises is high. Debt financing has a negative impact on enterprise performance.

### 5.2. Relationship between Debt Financing Structure and Enterprise Performance

**Figure 10:**
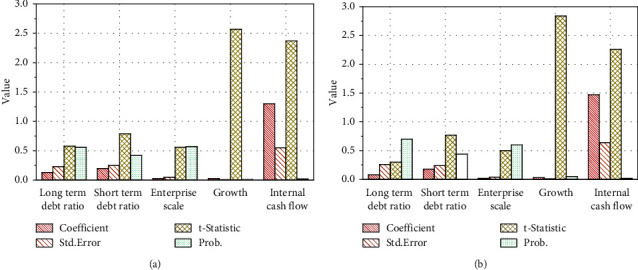
Multiple regression results of financing period on enterprise performance. (a) Individual fixed effect model. (b) Random effect model.

We establish the individual fixed effect model of debt financing term on enterprise performance. The specific multiple regression results are shown in Figure 10.

It can be seen from Figure 9 that the Hausman test result is 0.2145, which shows that there is not enough evidence to reject the original hypothesis at the 5% confidence level, that is, the impact of debt financing period on enterprise performance should choose to establish a random effect model. The explanation degree of the impact of debt financing term on enterprise performance is 51.29%. From the significance test, the adjoint probability of F-statistic of the regression equation is 0. At the importance level of 5%, the model is significant as a whole, the variables are linearly correlated, the three selected control variables are positively correlated with the explained variables, and the asset scale variable fails to pass the significance test, and the other two control variables passed the significance test at the 5% confidence level. The regression coefficient of long-term debt ratio is −0.089986, and the test value with significant coefficient difference is 0.7362. The long-term debt ratio is negatively correlated with the enterprise performance, but it is not significant; rejecting the hypothesis, the regression coefficient of short-term debt ratio is 0.188074, and the test value with significant coefficient difference is 0.4415. The short-term debt ratio is positively correlated with enterprise performance, but it is not significant. The average proportion of long-term debt is only 17.70%, while the average proportion of short-term debt is 82.30%. The unreasonable debt financing term structure makes the impact of long-term debt and short-term debt on enterprise performance not significant. More short-term debt does not match the capital operation of the enterprise and cannot effectively improve the company's performance.

## 6. Conclusion

A reasonable financing structure can reduce the financing cost and risk of enterprises and then help to improve the performance of enterprises. Therefore, the study of financing structure is of great significance to the development of enterprises. Taking the enterprise financing structure as the starting point, this paper generally grasps the current situation of the enterprise financing structure and enterprise performance assisted by blockchain for Internet of things supply chain financing mode. Based on the theoretical and empirical evaluation of financing forms and corporate performance, and integrated into the scenario of modern enterprises, this paper analyzes the equity financing structure from three aspects: equity financing structure and debt financing structure. Aiming at the Internet of things supply chain, this paper puts forward some suggestions on optimizing the enterprise financing structure with the help of the blockchain financing mode.

## Data Availability

The data used to support the findings of this study are available from the corresponding author upon request.
